# Bcl-xL-mediated remodeling of rod and cone synaptic mitochondria after postnatal lead exposure: Electron microscopy, tomography and oxygen consumption

**Published:** 2012-12-20

**Authors:** Guy A. Perkins, Ray Scott, Alex Perez, Mark H. Ellisman, Jerry E. Johnson, Donald A. Fox

**Affiliations:** 1 National Center for Microscopy and Imaging Research, University of California San Diego, La Jolla, CA; 2 Department of Natural Sciences, University of Houston-Downtown, Houston, TX; 3 College of Optometry, University of Houston, Houston, TX; 4 Department of Biology and Biochemistry, University of Houston, Houston, TX; 5 Department of Pharmacology and Pharmaceutical Sciences, University of Houston, Houston, TX

## Abstract

**Purpose:**

Postnatal lead exposure produces rod-selective and Bax-mediated apoptosis, decreased scotopic electroretinograms (ERGs), and scotopic and mesopic vision deficits in humans and/or experimental animals. Rod, but not cone, inner segment mitochondria were considered the primary site of action. However, photoreceptor synaptic mitochondria were not examined. Thus, our experiments investigated the structural and functional effects of environmentally relevant postnatal lead exposure on rod spherule and cone pedicle mitochondria and whether Bcl-xL overexpression provided neuroprotection.

**Methods:**

C57BL/6N mice pups were exposed to lead only during lactation via dams drinking water containing lead acetate. The blood [Pb] at weaning was 20.6±4.7 µg/dl, which decreased to the control value by 2 months. To assess synaptic mitochondrial structural differences and vulnerability to lead exposure, wild-type and transgenic mice overexpressing Bcl-xL in photoreceptors were used. Electron microscopy, three-dimensional electron tomography, and retinal and photoreceptor synaptic terminal oxygen consumption (QO_2_) studies were conducted in adult control, Bcl-xL, lead, and Bcl-xL/lead mice.

**Results:**

The spherule and pedicle mitochondria in lead-treated mice were swollen, and the cristae structure was markedly changed. In the lead-treated mice, the mitochondrial cristae surface area and volume (abundance: measure correlated with ATP (ATP) synthesis) were decreased in the spherules and increased in the pedicles. Pedicles also had an increased number of crista segments per volume. In the lead-treated mice, the number of segments/crista and fraction of cristae with multiple segments (branching) similarly increased in spherule and pedicle mitochondria. Lead-induced remodeling of spherule mitochondria produced smaller cristae with more branching, whereas pedicle mitochondria had larger cristae with more branching and increased crista junction (CJ) diameter. Lead decreased dark- and light-adapted photoreceptor and dark-adapted photoreceptor synaptic terminal QO_2_. Bcl-xL partially blocked many of the lead-induced alterations relative to controls. However, spherules still had partially decreased abundance, whereas pedicles still had increased branching, increased crista segments per volume, and increased crista junction diameter. Moreover, photoreceptor and synaptic QO_2_ were only partially recovered.

**Conclusions:**

These findings reveal cellular and compartmental specific differences in the structure and vulnerability of rod and cone inner segment and synaptic mitochondria to postnatal lead exposure. Spherule and pedicle mitochondria in lead-exposed mice displayed complex and distinguishing patterns of cristae and matrix damage and remodeling consistent with studies showing that synaptic mitochondria are more sensitive to Ca^2+^ overload, oxidative stress, and ATP loss than non-synaptic mitochondria. The lead-induced decreases in QO_2_ likely resulted from the decreased spherule cristae abundance and smaller cristae, perhaps due to Bax-mediated effects as they occurred in apoptotic rod inner segments. The increase in pedicle cristae abundance and CJ diameter could have resulted from increased Drp1-mediated fission, as small mitochondrial fragments were observed. The mechanisms of Bcl-xL-mediated remodeling might occur via interaction with formation of CJ protein 1 (Fcj1), whereas the partial protection of synaptic QO_2_ might result from the enhanced efficiency of energy metabolism via Bcl-xL’s direct interaction with the F1F0 ATP synthase and/or regulation of cellular redox status. These lead-induced alterations in photoreceptor synaptic terminal mitochondria likely underlie the persistent scotopic and mesopic deficits in lead-exposed children, workers, and experimental animals. Our findings stress the clinical and scientific importance of examining synaptic dysfunction following injury or disease during development, and developing therapeutic treatments that prevent synaptic degeneration in retinal and neurodegenerative disorders even when apoptosis is blocked.

## Introduction

Photoreceptor synapses are among the most complex in the mammalian nervous system. They have been the focus of several studies linking normal and pathophysiological structure to function [1-5]. The presynaptic terminals of rod and cone photoreceptors differ markedly from conventional synaptic terminals since the presynaptic terminals possess highly specialized and energetically active ribbon synapses that provide rapid, synchronous, and sustained glutamate release in the dark [6,7]. These synapses require mitochondria with densely packed cristae that produce high levels of ATP (ATP), as the mitochondria participate in the function and organization of synaptic vesicle pools, vesicular glutamate uptake and release, and temporal and spatial buffering of Ca^2+^ [8-11].

Photoreceptor apoptosis and visual deficits occur with inherited and toxicant-induced retinal degeneration, diseases, and aging [12-14]. One such neurotoxicant is inorganic lead. Children, non-human primates, and rodents with developmental lead exposure have cognitive, auditory, retinal, and visual-motor deficits [14-30]. Postnatal-only lead exposure produces rod-selective apoptosis characterized by a mitochondrial permeability transition, a subnormal scotopic electroretinogram (ERG) in mice and rats, and decreased dark-adapted rod photoreceptor oxygen consumption (QO_2_) [16,18,28,29]. Rod inner segment (RIS) mitochondria appear to be the primary site of action as cone inner segment (CIS) mitochondria were not affected by postnatal lead exposure [16-18,28,29,31]. However, the mitochondria in the presynaptic terminals of retinal photoreceptors, rod spherules and cone pedicles, were not examined after lead exposure. These studies also will provide unique comparisons of rod spherule and cone pedicle mitochondria to RIS and CIS mitochondria in similarly lead-exposed mice [29,32].

Chronic lead exposure during development and adulthood or in vitro lead exposure disrupted hippocampal N-methyl-D-aspartate (NMDA) presynaptic receptor formation, neurotransmitter release and plasticity [33,34] as well as decreased the expression of the outer mitochondrial membrane (OMM) voltage-dependent anion channel in auditory neurons and PC-12 cells [35,36]. Moreover, acute lead exposure decreased the number of synaptic vesicles and disrupted the structure of synaptosomal mitochondria isolated from the adult rat forebrain [37], consistent with the preferential subcellular localization of lead to mitochondria [38] and especially the inner mitochondrial membrane (IMM) and matrix fractions [39,40]. In consonance with the above findings, the current experiments had three specific aims. The first aim was to determine whether postnatal lead exposure affected spherule and/or pedicle mitochondria ultrastructure and cristae substructure. The second aim was to determine the functional effects of postnatal lead exposure on dark-adapted and light-adapted outer retinal (photoreceptor) QO_2_ and on dark-adapted photoreceptor synaptic QO_2_ using improved and newly developed procedures. The third aim was to determine whether Bcl-xL overexpression in photoreceptors, an antiapoptotic Bcl-2-like protein that blocked lead-induced rod selective apoptosis [29], mitigated or blocked the effects of postnatal lead exposure on spherule and pedicle mitochondria structure and function.

Electron tomography (ET) is the primary imaging tool for visualizing the three-dimensional internal structure of mitochondria, especially the cristae, OMM, and IMM [32,41,42]. ET has revealed new cellular and subcellular information, compared to conventional transmission electron microscopy (TEM), and revolutionized modern understanding of mitochondrial structure. A prime example is a revolutionary change in our understanding of crista and crista junction (CJ) organization and plasticity/remodeling [1,32,43-52]. Experiments in isolated liver mitochondria or cultured cells showed that tBid-mediated cytochrome c release and apoptosis resulted from CJ remodeling associated with the disassembly of IMM optic atrophy 1 (Opa1) protein complexes, although the underlying mechanisms have not been resolved [50,53,54]. Thus, we employed TEM and ET to investigate the ultrastructural, substructural, and three-dimensional organization of spherule and pedicle mitochondria in wild-type and Bcl-xL transgenic mice following postnatal lead exposure.

Our previous studies of RIS and CIS mitochondria [29,32] as well as spherule and pedicle mitochondria using ET [1] provided us with structural landmarks for comparison with lead-exposed, Bcl-xL transgenics, and lead-exposed Bcl-xL transgenics (Bcl-xL/lead) mitochondria. Confocal microscopy studies revealed that rod spherule membranes uniformly and intensely expressed the high affinity/low turnover plasma membrane Ca^2+^-ATPase, whereas the larger cone pedicles preferentially expressed isoform 1 of the low affinity/high turnover Na^+^-Ca^2+^ exchanger at their active zones [1]. TEM and ET established that mitochondria in rod spherules and cone pedicles differed markedly in number, location, size, volume and total cristae surface area, and cristae junction diameter. Rod spherules had one large ovoid mitochondrion located near the ribbon synapse, whereas cone pedicles averaged five medium-sized mitochondria clustered far from their active zones [1]. From these results as well as synaptic Ca^2+^ imaging studies in dark- and light-adapted retinas, we concluded that ATP (ATP) demand and mitochondrial ATP production were greater in cone pedicles than rod spherules.

In this study, we present novel results that reveal structural and functional information about the ability of spherule and/or pedicle mitochondria to remodel following postnatal lead exposure and the extent to which Bcl-xL overexpression protects against lead-induced alterations. Delineating the forms and mechanisms of mitochondrial remodeling is important and significant for determining their regenerative capacity, multiple roles in synaptic function, and roles in neurodegeneration and neuroprotection. Specifically, we show lead-induced mitochondrial remodeling in the form of mitochondrial matrix swelling, vesiculated cristae, and mitochondrial fragmentation as well as altered cristae abundance (i.e., cristae surface area/mitochondrial surface area and cristae volume/mitochondrial volume), cristae branching, and CJ opening. For spherules, Bcl-xL overexpression blocked most of the lead-induced mitochondrial remodeling except the cristae surface area that remained significantly lower. In contrast and unexpectedly, except cristae abundance and CJ density, the lead-induced alterations of pedicle cristae were not blocked by Bcl-xL overexpression. Moreover, Bcl-xL overexpression only partially protected against the lead-induced decrease in dark- and light-adapted photoreceptor QO_2_ and in dark-adapted photoreceptor synaptic QO_2_. To assess inter-compartmental differences, the results are compared with our RIS and CIS findings [29,40], which provides a wealth of important comparative information. The implications of mitochondrial remodeling in relation to apoptosis, mitophagy, and Bcl-xL overexpression as well as the possibility of long-term functional vision deficits in lead-exposed children are discussed. Moreover, the findings have clinical and scientific relevance for long-term synaptic dysfunction following injury or disease during development, and for developing effective therapeutic strategies and treatments that prevent synaptic degeneration in retinal degenerative/neurodegenerative disorders even if photoreceptor/neuronal apoptosis is prevented.

## Methods

### Materials

All chemicals were purchased as analytical or molecular biology grade from Sigma Chemical Co. (St. Louis, MO) or Fisher Scientific (Pittsburgh, PA), unless otherwise noted, and were certified lead-free (<5 ppb Pb^2+^). The pH of all solutions was 7.40 at the indicated temperatures.

### Experimental animals

All experimental and animal care procedures complied with the National Institutes of Health Public Health Service Policy on the Humane Care and Use of Laboratory Animals (NIH 2002) and were approved by the Institutional Animal Care and Use Committee of the University of Houston. Wild-type C57BL/6N mice (Harlan Sprague-Dawley, Indianapolis, IN), from litters bred at our facility, were maintained on a 12 h:12 h light-dark cycle (10–20 lux cage luminance) with food and water available ad libitum. Although these C57BL/6N mice had the rd8 mutation [55,56], we did not observe any patchy dysplasia, irregular variations in the thickness of any retinal layer, or displacement of photoreceptor nuclei into the inner nuclear layer in the inferior or superior retina of any mouse eye during development or at the time of our experiments, which is consistent with our previous studies [1,21,22,29,31,32]. As described [29], C57BL/6N Bcl-xL transgenic female mice were mated with control males. Pregnant mice were divided into control and lead-exposed groups. On the day of birth (postnatal day 0: P0) through weaning (P21), dams in the lead group received a 0.015% lead acetate drinking solution. Weaned pups received tap water and Purina chow ad libitum. All pups exhibited normal development. Animals were genotyped and divided into four groups: wild-type (control), Bcl-xL transgenic (Bcl-xL), wild-type/lead (lead), and Bcl-xL/lead.

For two reasons, adult (P60-P70) control and lead-exposed mice were used for all experiments. First, at this age the blood and retinal lead concentrations [Pb] were not different between groups. Our aim was to determine the long-term structural and functional effects of early postnatal lead exposure in the absence of elevated tissue lead. Blood and retinal [Pb] were determined using anodic stripping voltammetry (LeadCare® Kit1: sensitivity £1 µg/dl; Environmental Sciences Associates, Inc., Chelmsford, MA) and atomic absorption spectrometry as described [19,29]. At P21, mean ± standard error of the mean (SEM) blood and retinal [Pb] in the controls were 1.9±1.0 µg/dl and 0.02±0.02 mg/l, respectively, and in the lead-exposed mice were 20.6±4.7 µg/dl and 0.23±0.04 mg/l, respectively. At P60, mean ± SEM blood and retinal [Pb] in the controls were 3.6±1.8 µg/dl and 0.05±0.02 mg/l, respectively, and in the lead-exposed mice were 5.6±2.7 µg/dl and 0.09±0.04 mg/l, respectively. Second, our aim was to determine the effects of developmental lead exposure on synaptic mitochondria following significant turnover. Although we found no reports on mitochondrial fission and fusion in photoreceptor presynaptic mitochondria, the estimated half-life of total protein and RNA turnover in the cortical presynaptic mitochondria of young adult rats is 20 and 12 days, respectively [57]. Based on these results, ≤20% of the total mitochondrial protein and RNA present at P21 (end of lead exposure) will be present at P60-P70.

### Conventional transmission electron microscopy

We used our well validated fixation and embedding procedures that are optimal for maintaining photoreceptor mitochondria ultrastructure and substructure, as described [1,16,29]. Briefly, P60-P70 female mice were decapitated 2 h after light onset, the eyes were rapidly removed and immersed in ice-cold PBS (mono- and dibasic phosphate-buffered saline: 310 mOsm), the corneas were slit, and the eyes were immersion-fixed in ice-cold 3% glutaraldehyde, 2% paraformaldehyde, and 0.1% CaCl_2_ in 0.1 M cacodylate buffer (Karnovsky’s fixative) for 12 h at 4 °C. For three reasons, a piece of the central superior temporal retina located 200–250 μm from the optic nerve was used in each TEM and ET study. First, we used the same area of the mouse retina for our previous confocal, TEM, and ET work on mouse retinas [1,29,32], enabling a direct comparison of our RIS and CIS results to the current study on rod and cone synaptic terminal mitochondria. Second, this region contains mostly middle wavelength-sensitive M cones that are similar to those in other mammals [58,59], which enables cross-species comparisons. Third, this region does not exhibit any of the observed rd8 histopathological changes, especially at this age [55,56]. Ultrathin vertical sections of the retina were stained with uranyl acetate and lead citrate before being examined in a JEOL 100-C or 1200EX transmission electron microscope (Tokyo, Japan). Three to five different grids from five different control and lead-exposed mice were used.

### Three-dimensional electron microscope tomography

Mouse retinas were prepared for ET as described [1,29]. Briefly, the central superior temporal retinas of P60-P70 mice was trimmed (vide supra), and the Karnovsky-fixed retinal sections were dehydrated, embedded in Durcupan resin, sectioned (500 nm thick), and imaged using the single- and double-tilt series techniques as described [1,29,32]. Three to six mice from different litters and treatment groups were used for each experiment.

Intermediate voltage ET generates three-dimensional volumes of structures within the retinal tissue section at a z-axis resolution of 7–10 nm. The tomographic volumes are reconstructed by a back-projection algorithm from an area imaged with a wide range of tilt angles. The computer sections created in such a back-projected volume are typically 1–2 nm thick. Measurements were conducted on tomographic reconstructions from three rod spherules and three cone pedicles from each of three different mice from different litters (control, Bcl-xL, lead, and Bcl-xL/lead: 12 mice total) on equally spaced slices through their volumes. The reconstructed portion of each mitochondrion was segmented along the OMM, the inner boundary membrane (IBM), and the cristae membranes. The visualization tool, Synuview, was used to count the number of crista segments, and the programs Synuarea and Synuvolume calculated the surface area and volume values, respectively, for the OMM and each crista. A crista segment is defined as a portion of a crista, whether tubular or lamellar, with no sharp bends or branchings [44]. ImageJ (NIH) was used to measure the CJ diameter and CJ density.

### Photoreceptor and synaptic terminal oxygen consumption

For the light- and dark-adapted experiments, P60-P70 male and female mice were decapitated 2 h after light onset, or they were dark-adapted overnight and decapitated 2 h after scheduled light onset under dim red light (λ >650 nm). Whole retina, outer retina, and inner retina QO_2_ was recorded from isolated pairs of dark-adapted and light-adapted control, Bcl-xL, lead, and/or Bcl-xL/lead mouse neural retinas using our published dissection, stimulation, and recording procedures [29,60,61]. That is, QO_2_ was determined polarographically with a Clark-type oxygen electrode in a water-jacketed and temperature controlled Gilson-type oxygen chamber using calibrated white light delivered by a fiber optic. However, a modified outer-inner retina pharmacological isolation buffer that contained more specific glutamate antagonists was used. The previous buffer contained 100 µM of 2-amino-4-phosphonobutyric acid (APB), a glutamate analog that binds mGluR6 receptors of ON bipolar cells [62], and 5 mM kynurenic acid, a non-competitive antagonist of NMDA and kainate receptors [63]. The buffer used in the present experiments contained 100 µM APB, 50 µM 6-cyano-7-nitroqunioxaline-2,3-dione (CNQX), or 2,3-dihydroxy-6-nitro-7-sulfamoylbenzo-quinoxaline (DNQX), competitive antagonists of α-amino-3-hydroxy-5-methyl-4-isoxazolepropionic acid (AMPA) and kainate receptors [64,65], and 100 µM D(-)-2-amino-5-phosphovalerate (APV), a competitive antagonist at NMDA receptors [64]. To isolate and determine the photoreceptor synaptic terminal QO_2_, 2 mM CoCl_2_ was added to the new isolation buffer. This concentration of CoCl_2_ blocks all photoreceptor glutamate release as no ERG b-wave is present, but does not affect the ERG a-wave [63,66]. In addition, the effects of 10 µM nifedipine, an L-type Ca^2+^ channel antagonist that blocks photoreceptor glutamate release but not the photoreceptor outer segment cyclic guanosine monophosphate-gated channel [67,68], on photoreceptor synaptic terminal QO_2_ was examined in the absence and presence of CoCl_2_. CoCl_2_ did not affect retinal Na^+^/K^+^-ATPase activity, retinal cyclic guanosine monophosphate-phosphodiesterase activity, or isolated retinal mitochondrial QO_2_ with glutamate-malate substrates (Fox, unpublished data). Retinas from four to seven mice from different litters and treatment groups were used for each experiment. No differences in male and female mice were observed for any condition or treatment, so the data were combined.

### Statistical analyses

For a given set of data, only one adult animal from a litter per treatment was used, and the values represent three to seven animals per treatment group. Data were analyzed with an ANOVA (ANOVA) followed by post-hoc multiple comparisons using Tukey's Honestly Significant Difference test. Data are presented as the mean±SEM. In graphs and tables, values with p<0.05 were considered significantly different from the controls or other comparison groups, and were noted where appropriate by superscript letters. Graphs were generated with KaleidaGraph 4.0 (Synergy Software, Reading PA).

## Results

### Conventional transmission electron microscopy

**Figure 1:**
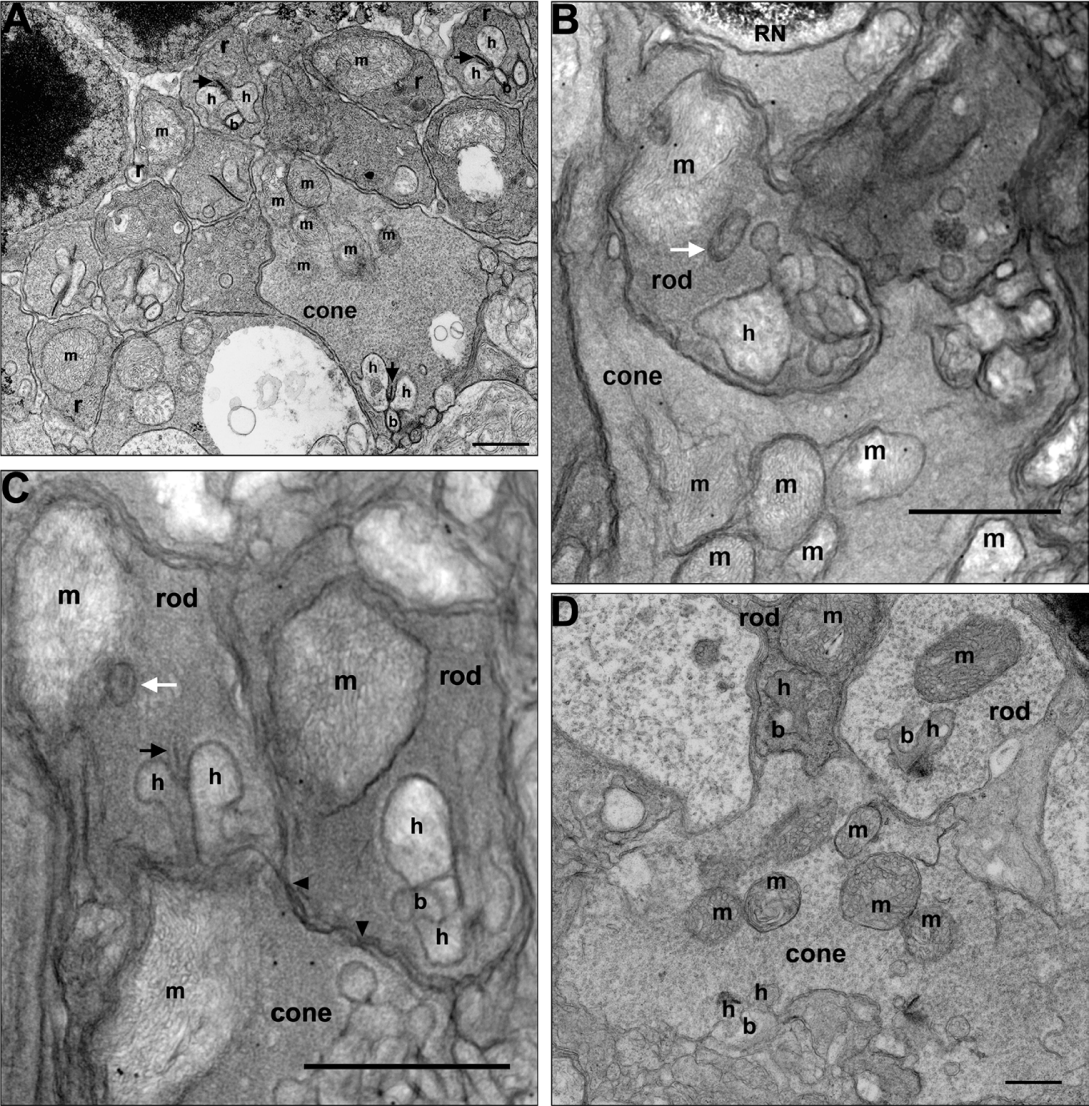
Altered structure of rod spherules and cone pedicles in adult mice following postnatal-only lead exposure. Postnatal developmental lead exposure alters the structure of rod spherules and cone pedicles in adult mice. **A**: A longitudinal section of a control retina showing several rod spherules and a cone pedicle. **B**: Longitudinal section showing two rod spherules and a cone pedicle from a lead-exposed mouse. A rod nucleus (RN) in the most proximal row of the outer nuclear layer is not retracted. The rod spherule of the left contains a moderately swollen mitochondrion (m) and a swollen cisterna of agranular endoplasmic reticulum (white arrow). One of the lateral horizontal cell processes (h) of the invaginating synapse is quite swollen. The rod spherule of the right contains a mildly swollen mitochondrion (m), and one lateral horizontal cell process (h) is quite swollen. A larger more electron lucent cone pedicle contains a moderately swollen mitochondrion. The invaginating processes of the lateral horizontal cells and a central bipolar cell also are swollen. Scale bar=1 micron. **C**: A longitudinal section from a different lead-exposed mouse showing two rod spherules and a cone pedicle. In the spherules and pedicles, the mitochondria and cisterna of the agranular endoplasmic reticulum (white arrow) adjacent to the mitochondrion (m) are often swollen. Similarly, the lateral horizontal cell processes (h) of the invaginating synapse can be swollen. A synaptic ribbon (black arrow) is visible. The larger more electron lucent cone pedicle appears to have swollen invaginating synapses. Gap junctions between the large cone pedicle and the adjacent rod spherule remain (solid black arrowheads). Scale bar=1 micron. **D**: A longitudinal section from a Bcl-xL/lead mouse showing a rod spherule and a cone pedicle. Bcl-xL overexpression blocked most of the lead-induced mitochondrial and neuronal dendritic process swelling. Scale bar=1 micron.

High magnification electron micrographs illustrate the cellular and organelle structures of adult control (Figure 1A), lead (Figure 1B,C), and Bcl-xL/lead (Figure 1D) rod spherules and cone pedicles. As previously described, control spherules form two to four tiers positioned above the larger more electron lucent pedicles. The spherules contain a single large ovoid mitochondrion located close to the synaptic ribbon complex, whereas each pedicle has four to six mitochondria located far from the synaptic ribbon complexes. The mitochondria in both synaptic terminals are in the orthodox conformation, which is characterized by a relatively large matrix volume and small intracristal space as the IBM is closely apposed to the OMM. The invaginating synapse of the rod and the 6–14 invaginating synapses in cones contain dendrites from two lateral horizontal cells and a central bipolar cell, although the rods have a longer synaptic ribbon compared to the cones [1]. In Bcl-xL mice, the ultrastructure of the spherule and pedicle and their mitochondria were not different from the controls (data not shown).

Figure 1B,C reveal typical examples of the lead-induced alterations in cellular and organelle structures observed in most spherule and pedicle synapses. The invaginating synapses of the spherules and pedicles, lateral horizontal cell processes and central bipolar cell process, and endoplasmic reticulum (white arrow) are mild to moderately swollen and distorted. The spherule and pedicle mitochondria display matrix swelling and sub-compartmentation. However, the OMM is not ruptured, the gap junctions between the spherules and pedicles (black arrowheads) are not disrupted, and the rod and cone nuclei in the most proximal outer nuclear layer are not retracted as typically seen in detached retinas [69]. Electron-dense lead granules were not observed in any mitochondria. Figure 1D reveals that Bcl-xL overexpression partially blocked many of the lead-induced synaptic and mitochondrial alterations, especially the mitochondrial and neuronal dendritic process swelling. However, spherule and pedicle mitochondria still have some vesiculated cristae and swollen matrix compartments. To fully and properly characterize the lead-induced structural alterations on these synaptic mitochondria and their cristae in the absence and presence of Bcl-xL overexpression, ET experiments were conducted.

### Electron microscope tomography

**Figure 2:**
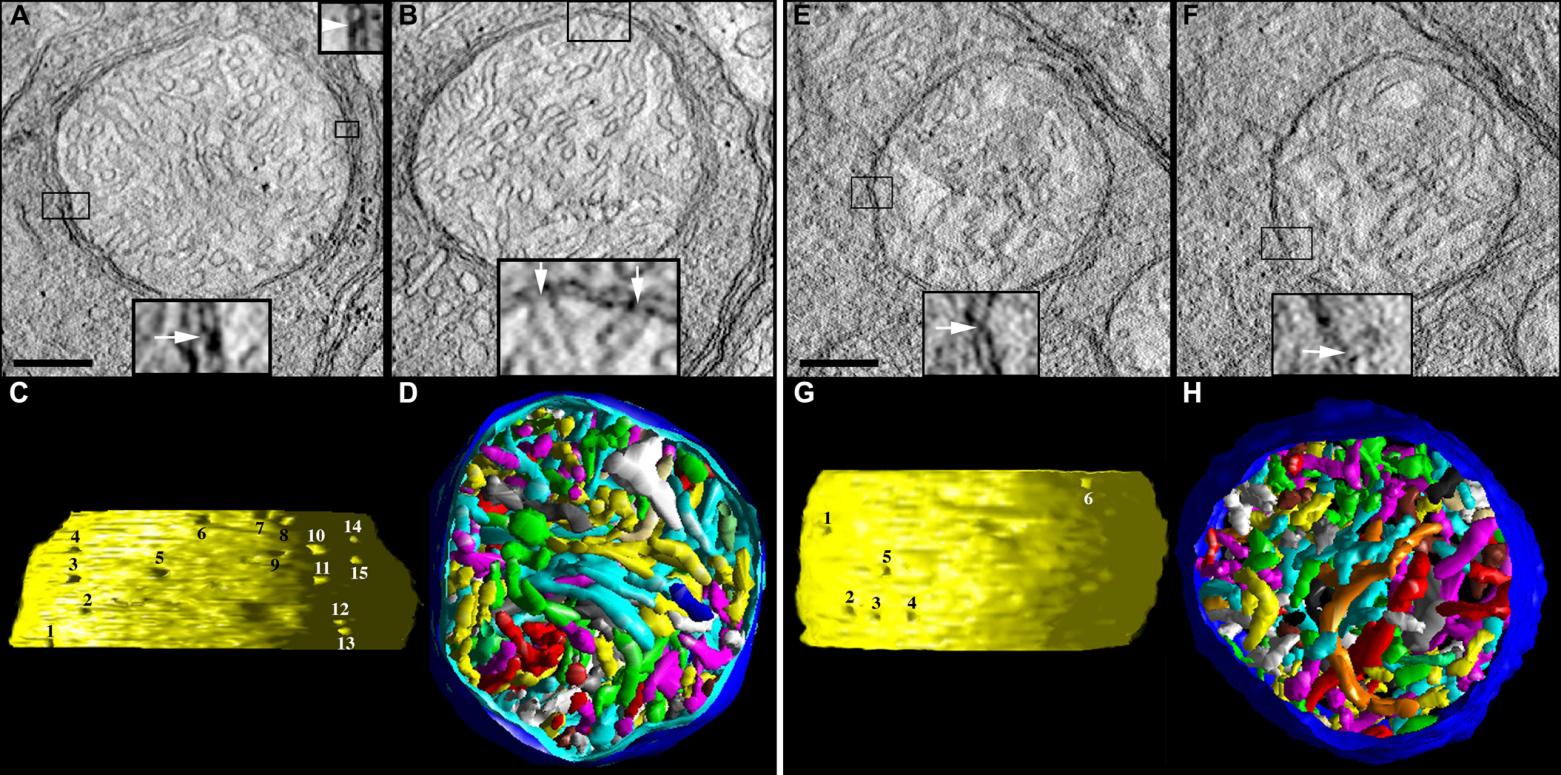
Control spherule and pedicle mitochondria possess many cristae, cristae with tubular and lamellar segments, narrow crista junctions, and punctate contact sites. **A**-**D**: Tomographic reconstructions of a rod spherule mitochondrion from an adult control mouse are presented. **A**: A 2.2 nm slice through the center of a tomographic volume of a spherule shows the membrane profiles and associations including those of the outer and inner boundary membranes and cristae in a large mitochondrion with many cristae. The inset at the bottom provides an example of a classic contact site (boxed and enlarged 3X), defined as the site where the outer mitochondrial membrane (OMM) and inner boundary membrane (IBM) are joined by pinching together (arrow). The inset at the top provides an example of a bridge contact site (boxed and enlarged 3X), defined as the site where the OMM and IBM are joined by a bridge or tether (arrowhead). Scale bars=200 nm. **B**: Another slice through the volume showing a crista junction (boxed), which is an opening, often tubular, connecting the intracristal space with the intermembrane space. The inset at the bottom shows the opening (arrows) of two adjacent crista junctions enlarged 3X. **C**: Side view of the inner membrane of the segmented and surface-rendered volume displayed with left lighting showing the size and density of crista junctions. Crista junction openings were found to be invariably narrow, tubular, and remarkably uniform in diameter. There are 15 numbered crista junction openings in this view. **D**: Top view of the segmented and surface-rendered volume showing the outer membrane (blue) and the entire complement of 170 cristae (various colors) provides a three-dimensional feel for the packing arrangement and density of the cristae. Most of the cristae are tubular. However, some cristae are completely lamellar or possess lamellar compartments attached to tubular compartments, and still connect to the intermembrane space via tubular crista junctions. Compare with Figure 7. **E**–**H**: Tomographic reconstructions of a cone pedicle mitochondrion from an adult control mouse are presented. **E**: A 2.2 nm slice through the center of a tomographic volume of a pedicle shows a medium-sized mitochondrion. The classic contact sites have the same structure as those in the spherule mitochondria; an example contact site is boxed and shown enlarged 3X in the inset. **F**: Another slice through the volume showing a crista junction (boxed), enlarged 3X in the inset. **G**: Side view of the inner membrane of the segmented and surface-rendered volume. The crista junction architecture was found to be similar to that in the spherule mitochondria. There are six numbered crista junction openings in this view. **H**: Top view of the segmented volume showing the outer membrane (blue) and the entire complement of 165 cristae (various colors). As with spherule mitochondria, most of the cristae are tubular. However, some cristae are lamellar or possess lamellar compartments. Compare with Figure 8.

For comparison with the spherule and pedicle mitochondria from the lead, Bcl-xL, and Bcl-xL/lead groups, we present the salient ultrastructural and substructural features of wild-type control spherule and pedicle mitochondria (Figure 2). These features were analyzed first by Johnson and coworkers [1] and are further described through greater sampling and analysis. Figure 2 shows 2.2 nm thick slices through spherule (Figure 2A,B) and pedicle (Figure 2E,F) mitochondria made from ET reconstructions. The single ovoid mitochondrion in the spherules is larger than the four to six mitochondria in the pedicles. Volume segmentation was used to illustrate the cristae architecture. Control spherule and pedicle mitochondria possess many cristae that are branched with tubular and lamellar branching segments. Most cristae are tubular; however, some cristae are lamellar or possess lamellar compartments attached to tubular compartments, and still connect to the intermembrane space via tubular CJs. As is common among many classes of mitochondria, the CJs of the spherule and pedicle mitochondria are narrow tubes, and the contact sites that attach the OMM and IBM together are punctate.

**Figure 3:**
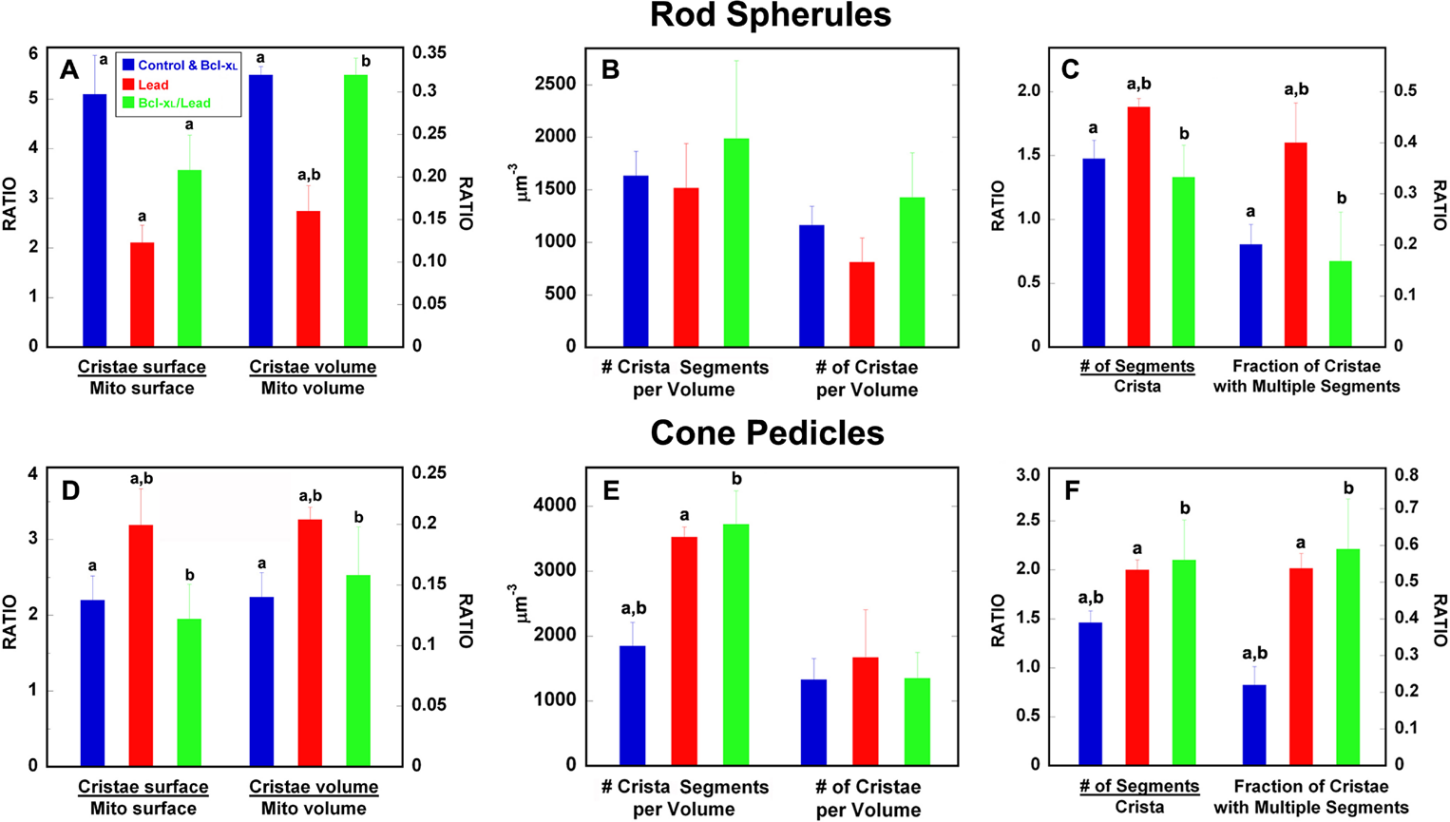
Quantitative comparison of rod spherule and cone pedicle mitochondrial cristae measurements in control, Bcl-xL, lead-exposed (Lead), and Bcl-xL and lead-exposed (Bcl-xL/Lead) mice obtained from tomographic reconstructions. The mean values for all mitochondrial measures in the control and Bcl-xL mice were not significantly different, so they were combined (blue bars). Data for the Lead (red bars) and Bcl-xL/Lead (green bars) mice are presented for each plot. **A**–**C**: Rod spherule mitochondria. **A**: The cristae surface area/mitochondrial surface and cristae volume/mitochondrial volume decreased in Lead. Bcl-xL overexpression (Bcl-xL/Lead) partially protected against the loss of cristae surface and fully protected against the loss of cristae volume. **B**: The number of crista segments and cristae per volume were not different in the Control/Bcl-xL, lead, or Bcl-xL/Lead mice. **C**: The number of segments per crista and the fraction of cristae with multiple segments increased in the Lead mice and were blocked in the Bcl-xL/Lead mice. **D**–**F**: Cone pedicle mitochondria. **D**: The cristae surface area/mitochondrial surface and cristae volume/mitochondrial volume increased in the Lead mice and were protected in the Bcl-xL/Lead mice. **E**: The number of crista segments increased in the Lead and Bcl-xL/Lead mice. The cristae per volume were not different in the Control/Bcl-xL, Lead, or Bcl-xL/Lead mice. **F**: The number of segments per crista and the fraction of cristae with multiple segments increased in the Lead and Bcl-xL/Lead mice. Values represent mean±SEM measurements from three to six different mice from different litters per treatment. Values sharing the same superscript differed from each other at p<0.05.

Figure 3 presents cristae measurements of the spherule and pedicle mitochondria obtained from tomographic reconstructions. The control spherule mitochondrion has a high cristae surface/mitochondrial surface area ratio (5.81), which is a measure of mitochondrial ATP synthesizing capacity that correlates with the QO_2_ [70]. This ratio is 2.64-fold greater than each pedicle mitochondrion (Figure 3A) and 3.40-fold greater than in retinal ganglion cell, brain, and spinal cord mitochondria [32]. For comparison, CISs, which possess twofold more mitochondria than RISs [32], have a moderate cristae surface/mitochondrial surface area ratio (2.60) that is 2.16-fold higher than in RIS mitochondria [32]. Moreover, CISs and cone pedicles have higher cytochrome oxidase and Na^+^/K^+^-ATPase activity than RISs and rod spherules [1,71,72]. Taken together and relevant for this investigation, we previously concluded that cones have higher ATP demand and mitochondrial ATP production than rods [1,32] and suggested that increased rod susceptibility/apoptosis occurs under conditions where low-to-moderate Ca^2+^ overload occurs in the absence of oxidative stress, while increased cone susceptibility/apoptosis occurs under circumstances where oxidative stress predominates [31].

**Figure 4:**
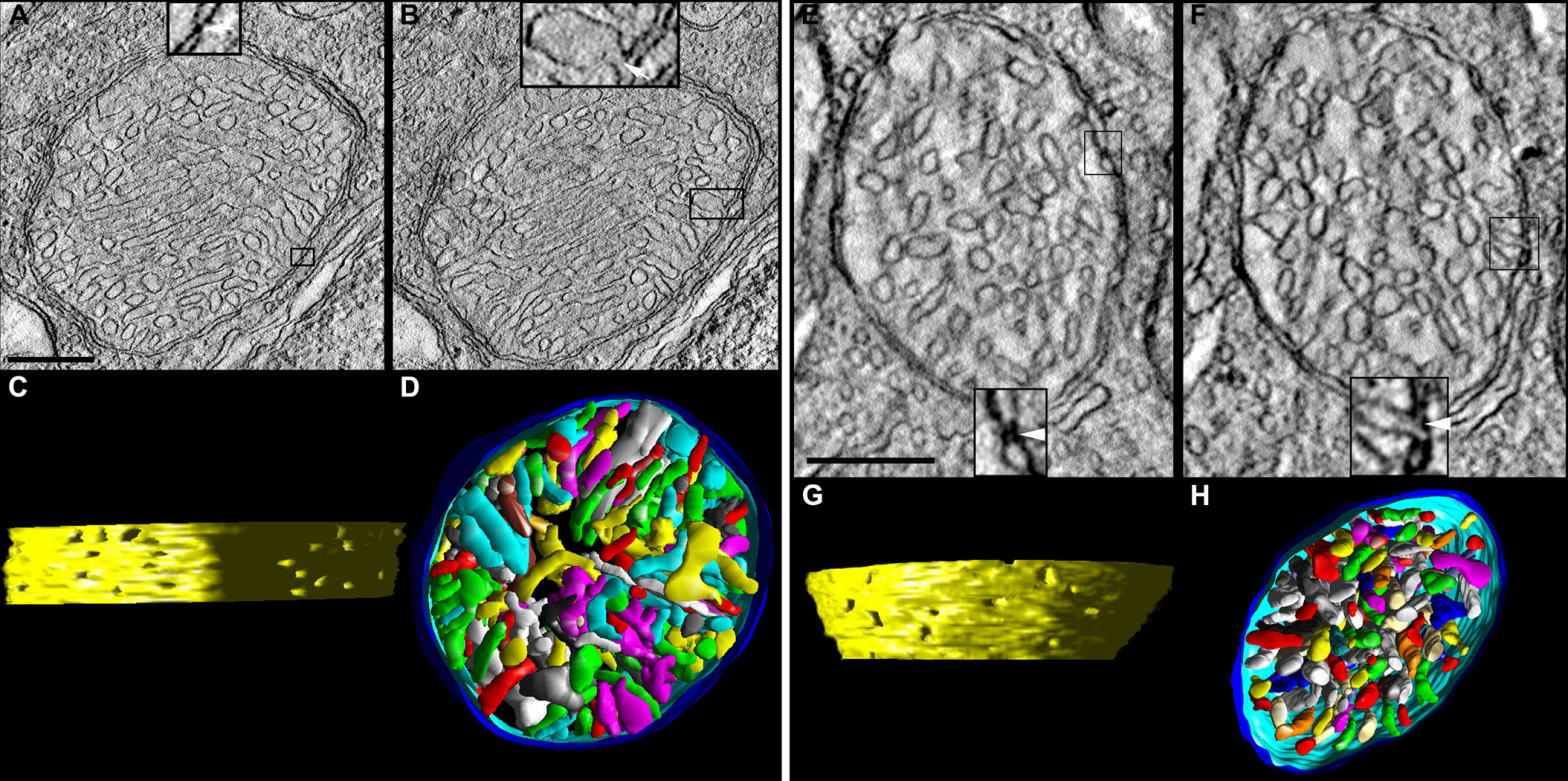
In the Bcl-xL transgenic mice, the structure of the spherule and pedicle mitochondria is similar to that of the control mice. **A**–**D**: Tomographic reconstructions of a Bcl-xL rod spherule mitochondrion from an adult mouse are presented. (**A** and **B**) The 2.2 nm slices near the middle of the volume show the dense packing of cristae typical for Bcl-xL and control mitochondria. The contact sites and crista junctions (insets) have the same architecture as their control counterparts. Scale bars=200 nm. **C**: The side view of the inner membrane of the segmented volume shows 21 crista junction openings in this view. The crista junction diameter is increased compared to controls; see Table 1. **D**: The top view of the segmented volume shows the outer membrane and the entire complement of 173 cristae (various colors). **E**–**H**: Tomographic reconstructions of a Bcl-xL cone pedicle mitochondrion from an adult mouse are presented. (**E** and **F**) The 2.2 nm slices near the middle show a smaller mitochondrion with densely packed cristae. The contact sites and crista junctions (insets) have the same architecture as their control and spherule Bcl-xL counterparts. **G**: The side view of the inner membrane of the segmented volume shows 13 crista junction openings in this view. **H**: The top view of the segmented volume showing the outer membrane and the entire complement of 110 cristae (various colors).

ET images and cristae measurements of spherule and pedicle mitochondria obtained from tomographic reconstruction revealed that Bcl-xL overexpression did not alter the basic ultrastructure or substructure of the spherule or pedicle mitochondria (Figure 3 and Figure 4). Quantitatively, there was no difference in the abundance of cristae or their segments between the control and Bcl-xL mitochondria, and therefore, their data were combined (Figure 3). The only notable difference was that the CJ diameter in the Bcl-xL spherule mitochondria significantly increased (+18%) compared to controls (Table 1). Interestingly, especially in relation to findings that changes to the CJ diameter may not correlate with cytochrome c release during apoptosis [reviewed in 40], apoptosis in the developing rods was completely blocked by Bcl-xL overexpression in the photoreceptors [29]. The CJ diameter in the control and Bcl-xL spherule mitochondria was significantly larger (41%–53%) than that in the pedicle mitochondria (Table 1).

**Table 1:** Mitochondrial crista junction diameter and density in rod spherules and cone pedicles: effects of postnatal lead exposure and/or bcl-xl overexpression.

Name	Control	Lead	Bcl-xL	Bcl-xL/Lead
Crista Junction Diameter (nm)				
Rod Spherule	12.00±0.39^a,b^	11.40±0.22^c,d^	14.10±0.34^a,c^	13.40±0.50^b,d^
Cone Pedicle	8.50±0.42^a,b^	10.90±0.60a	9.20±0.33^c^	10.30±0.40^b,c^

Crista Junction Density (number of CJs /mm^2^)				
Rod Spherule	54±7	74±17	58±8	48±20
Cone Pedicle	56±18	72±19	65±13	61±18

Values represent mean±SEM measurements from three to six different mice from different litters per treatment. For rod spherules, 85–103 cristae junctions were measured. For cone pedicles, 32–120 crista junctions were measured. Values sharing the same superscript differed from each other at p<0.05.

**Figure 5:**
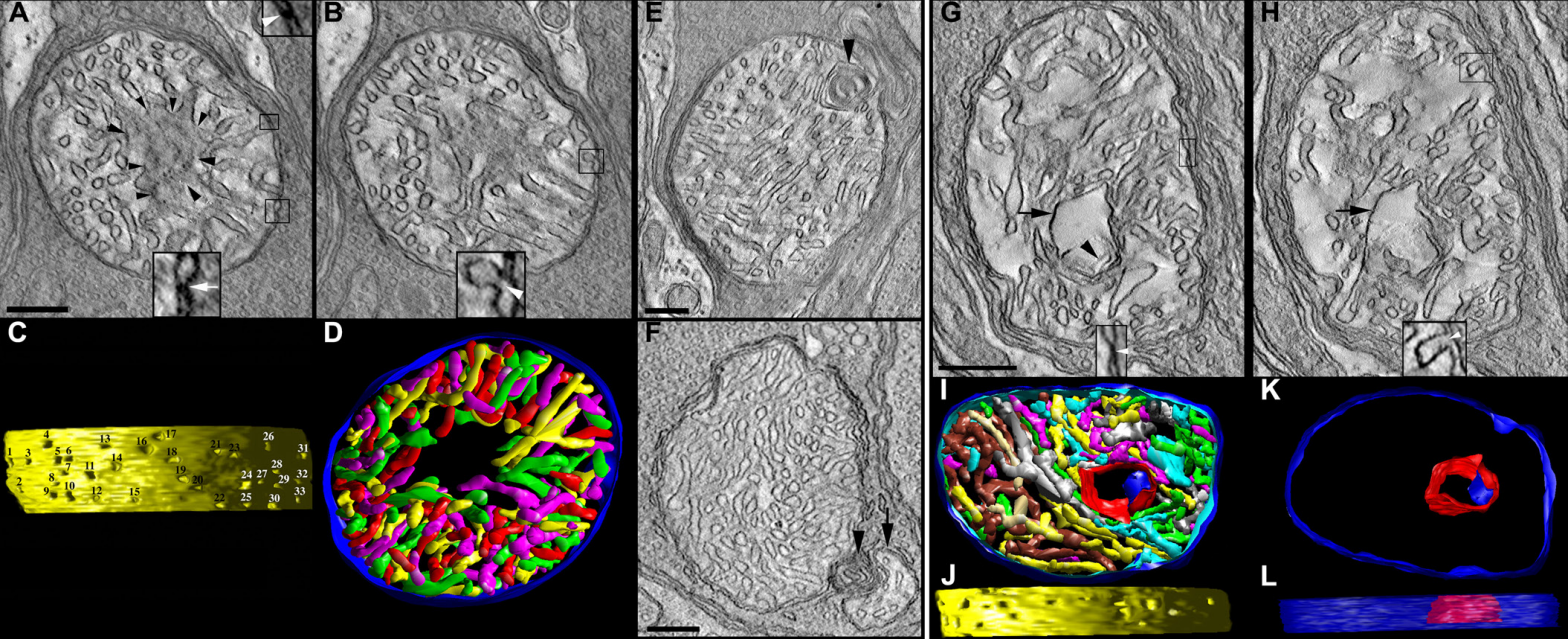
In the lead-exposed mice, the spherule and pedicle mitochondria have an altered cristae structure. **A**–**D**: Tomographic reconstructions of a rod spherule mitochondrion from a developmentally lead-exposed adult mouse are presented. **A**: A 2.2 nm slice through the middle shows a medium-sized mitochondrion with cristae missing especially in the center of the organelle (black arrowheads). The classic contact sites (arrow), bridge contact sites (arrowhead), and crista junctions (**B**) have the same architecture as their control counterparts. Scale bars=200 nm. **B**: Another slice through the volume shows the degraded cristae. **C**: The side view of the inner membrane of the segmented volume is displayed with left lighting. There are 33 numbered crista junction openings in this view. **D**: The top view of the segmented volume shows the outer membrane and the entire complement of 98 cristae (various colors). The region with missing cristae in the center of the mitochondrion extends throughout the reconstructed volume. **E**: Another feature of lead-exposed mitochondria is a form of inner membrane degeneration known as “swirls” or “myelin-like” (arrowhead). **F**: A separate mitochondrion displayed the swirl (arrowhead) between the main mitochondrial body and a small mitochondrial fragment (arrow) detached from the main body. **G**–**L**: Tomographic reconstructions of a cone pedicle mitochondrion from a developmentally lead-exposed adult mouse are presented. (**G** and **H**) The 2.2 nm slices near the middle show a medium-sized mitochondrion containing a large, abnormal vesiculated crista (black arrows) with a much smaller enclosed internal membrane (black arrowhead). As with the lead-exposed spherule mitochondrion, the contact sites and crista junctions (insets) have the same architecture as the controls. Scale bars=200 nm. **I**: The top view of the segmented volume shows the outer membrane and the entire complement of 69 cristae (various colors). **J**: The side view of the inner membrane of the segmented volume shows 16 crista junction openings in this view. The crista junction diameter is increased compared to the controls; see Table 1. **K**: The top view and (**L**) side view of the vesiculated crista (red) and internal membrane (blue) show the extent of the volume (outer membrane in translucent blue) occupied by this abnormal structure.

Figure 5 shows that lead exposure markedly changed the cristae structure of the spherule and pedicle mitochondria. The mitochondria often have sub-volumes of degenerated or missing cristae (Figure 5A,B,F-H) with vesicularization, as suggested by the electron micrographs (Figure 1B,C). In addition, the internal membranes exhibit degeneration characterized by “swirls” or “onion-like” formations. These striking rearrangements of the cristae often accompanied small mitochondrial fragments that were in the process of detaching or had already detached from the main mitochondrial body (Figure 5E,F). These small mitochondrial fragments may be destined for mitophagy, an elimination process observed in apoptotic cells [73,74] and partially mediated by the proteolytic processing of Opa1 [75]. Another common feature was mitochondria that contained one or more large, abnormal vesiculated crista (Figure 5G-I,K,L). Occasionally, inside these vesiculations a much smaller vesicle was observed. The IBM degeneration or remodeling did not significantly modify the number of contact sites (Figure 5C,J) or CJ density (Table 1), although the latter increased (29%–37%) in spherules and pedicles in lead-exposed mice. Moreover, in lead-exposed mice the CJ diameter in pedicle, but interestingly not spherule, mitochondria increased about 30% compared to the controls (Table 1), consistent with the idea that IBM remodeling is a component of apoptotic events [53,54].

Quantitative analyses of the spherule and pedicle mitochondria substructural features were performed and compared for the control and lead groups. Lead produced contrasting effects on cristae surface area and cristae volume in spherule compared to pedicle mitochondria, such that both measures significantly decreased in spherules (−64% and −50%, respectively; Figure 3A) and increased in pedicles (45% and 46%, respectively; Figure 3D). The number of crista segments per volume and the number of cristae per volume in spherule mitochondria were not affected by lead exposure (Figure 3B), whereas the number of crista segments per volume increased 1.91-fold in pedicles from the lead mice (Figure 3E). The number of segments per crista and fraction of cristae with multiple segments increased similarly and significantly in spherule mitochondria (27% and 110%, respectively; Figure 3C) and pedicle mitochondria (37% and 145%, respectively; Figure 3F).

**Figure 6:**
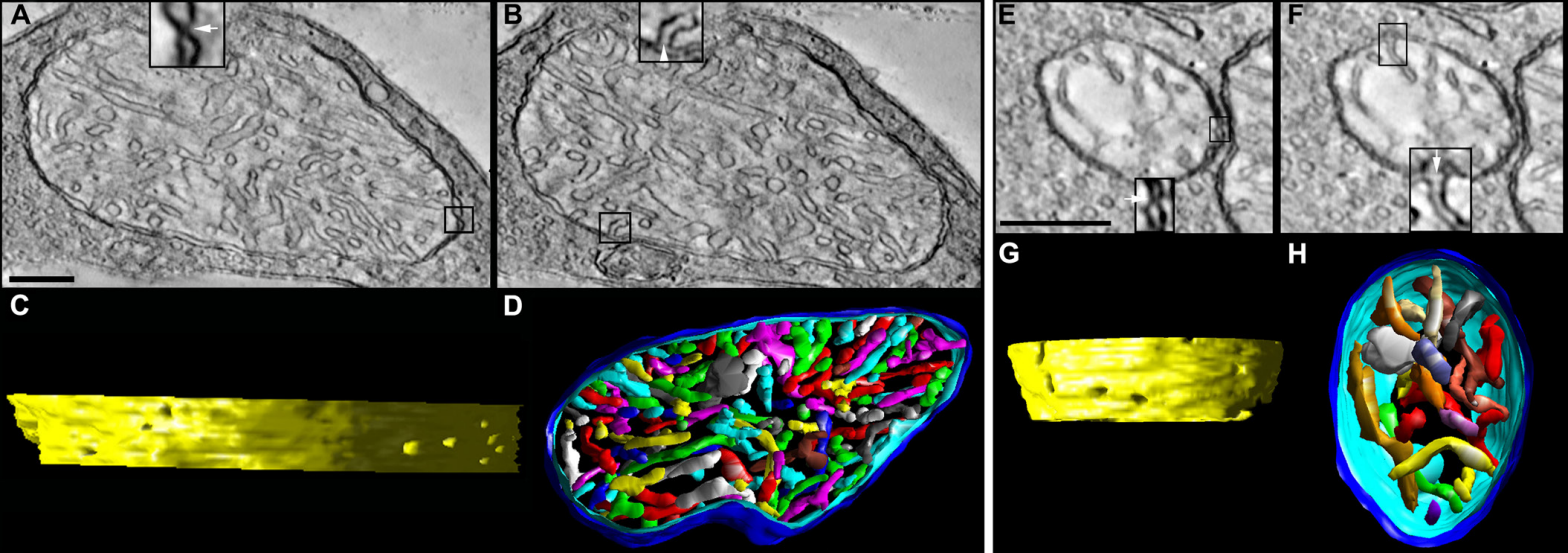
Bcl-xL does not fully protect spherule and pedicle mitochondria from postnatal lead exposure. **A**–**D**: Tomographic reconstructions of a Bcl-xL/Lead rod spherule mitochondrion from an adult mouse are presented. (**A** and **B**) The 2.2 nm slices through the middle of the volume show a large mitochondrion with fewer cristae compared to the control and Bcl-xL and with contact sites and crista junctions (insets) with the same architecture as their control counterparts. Scale bars=200 nm. **C**: The side view of the inner membrane of the segmented volume is displayed with left lighting. There are nine numbered crista junction openings in this view. The crista junction diameter is increased relative to the lead-exposed and control mitochondria; see Table 1. **D**: The top view of the segmented volume shows the outer membrane and the entire complement of 97 cristae (various colors). The cristae packing density is noticeably less than in the control or Bcl-xL mitochondria (compare to Figure 3 and Figure 4). **E**–**H**: Tomographic reconstructions of a Bcl-xL/Lead cone pedicle mitochondrion from an adult mouse are presented. (**E** and **F**) The 2.2 nm slices near the middle show a small mitochondrion. The contact sites and crista junctions (insets) have the same architecture as the control, Bcl-xL, and lead. Scale bars=200 nm. **G**: The side view of the inner membrane of the segmented volume shows eight crista junction openings in this view. **H**: The top view of the segmented volume shows the outer membrane and the entire complement of 18 cristae (various colors). The shape and size of the cristae are similar to that in the control and Bcl-xL mitochondria (compare to Figure 3 and Figure 4). However, the crista density is significantly less than in the lead-exposed mitochondrion (compare to Figure 5).

Bcl-xL overexpression partially and differentially protected the spherule or pedicle mitochondria from the lead-induced cristae alterations (Figure 3 and Figure 6; Table 1 and Table 2). This is in contrast to our findings that Bcl-xL completely blocked the lead-induced rod-selective apoptosis mediated by RIS mitochondria [29]. As illustrated in Figure 1D and Figure 6, the Bcl-xL/lead spherule and pedicle mitochondria had decreased matrix swelling and cristae vesicularization, although they still had missing cristae (Figure 6A, B,D-F,H; Figure 1D). Overall, the protection appeared greater in spherule than in pedicle mitochondria (Figure 3; Table 1 and Table 2). In the spherule mitochondria, Bcl-xL partially blocked the lead-induced decrease in the cristae surface area (Figure 3A), whereas Bcl-xL completely blocked the decreased cristae volume (Figure 3A), increased number of segments per crista (Figure 3C), and increased fraction of cristae with multiple segments (Figure 3C); also see Table 2. In the pedicle mitochondria, Bcl-xL completely blocked the lead-induced increase in the cristae surface area and cristae volume (Figure 3D). In contrast, in the pedicle mitochondria Bcl-xL did not block the increased number of crista segments per volume (Figure 3E), number of segments per crista (Figure 3F), fraction of cristae with multiple segments (Figure 3F), or CJ diameter (Table 1); also see Table 2. Taken together, the three-dimensional reconstructions and quantitative results demonstrate that postnatal lead exposure altered the structure of the rod and cone synaptic mitochondrial cristae and that Bcl-xL overexpression provided greater structural protection for the spherule than the pedicle mitochondria.

**Table 2:** Bcl-xl-mediated protection of lead-induced substructural alterations in rod spherule and cone pedicle mitochondria.

Mitochondrial substructural measure	Rod spherule		Cone pedicle	
	Lead	Bcl-xL/Lead	Lead	Bcl-xL/Lead
Cristae surface/Mito surface	↓↓	↓	↑↑	±
Cristae volume/Mito volume	↓↓	±	↑↑	±
# Crista segments/Volume	±	±	↑↑↑	↑↑↑
# Cristae/Volume	±	±	±	±
# Segments/Crista	↑	±	↑↑	↑↑
Fraction of cristae with multiple segments	↑↑↑	±	↑↑↑	↑↑↑
Crista junction diameter	±	±	±	±
Crista junction density	±	±	±	±

Arrows indicate relative significant percent change from controls as follows: 1 arrow=≤±35%; 2 arrows=±36%–89%; 3 arrows=≥±90%. The symbol ±=not significantly different from controls.

**Figure 7:**
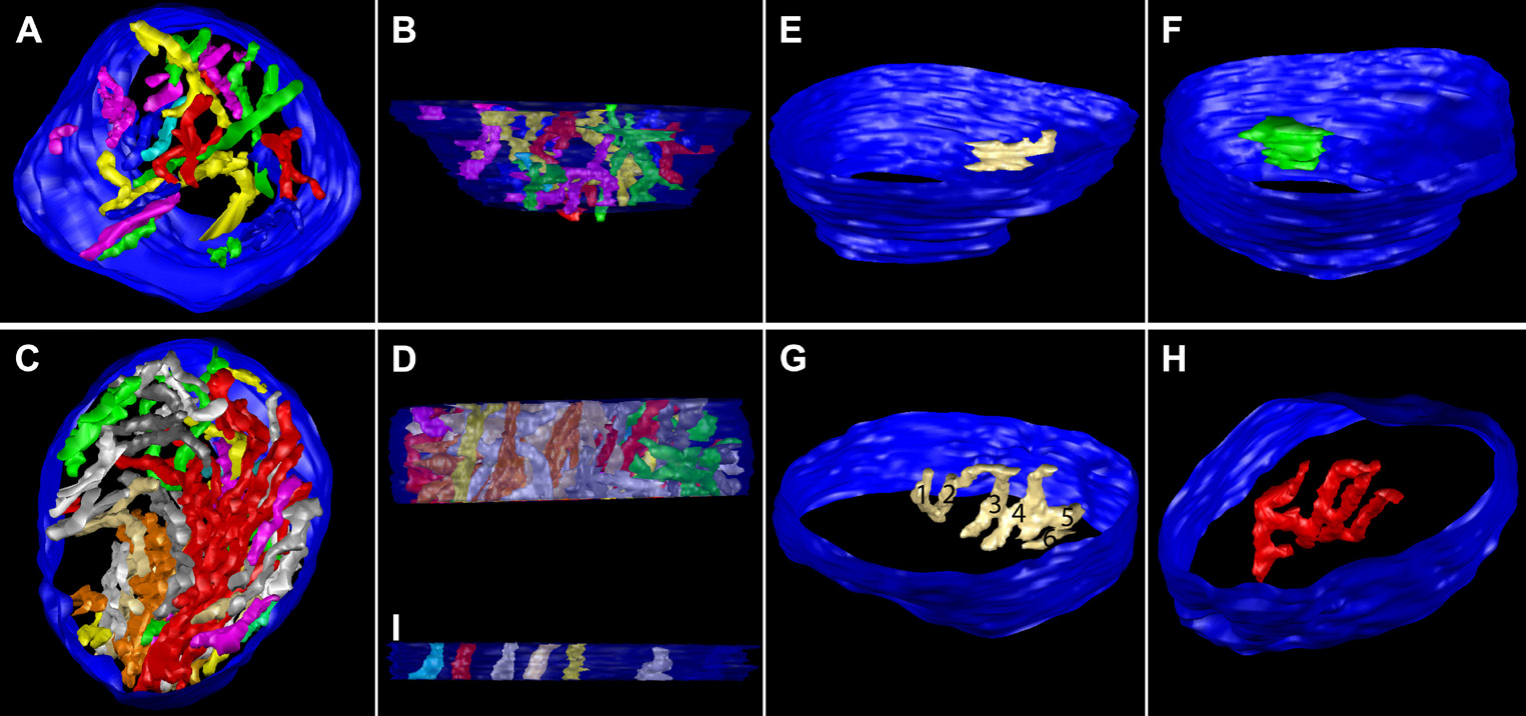
Lead-exposed spherule mitochondria have more cristae with multiple segments than the controls. (**A**) The top and (**B**) side views display only cristae with multiple segments (30 out of 204 cristae) in a control spherule mitochondrion. The outer membrane is shown in blue. **C**: The top and (**D**) side views show only cristae with multiple segments (33 out of 98) in a lead-exposed spherule mitochondrion. Note the much greater volume of mitochondrion occupied by cristae with multiple segments in the lead spherule. Examples of typical lamellar cristae in (**E**) control and (**F**) lead mitochondria. As is common with lamellar cristae in terminal mitochondria, they are small and do not extend far across the volume. These are examples of typical cristae with multiple segments in (**G**) control and (**H**) lead mitochondria. Crista segments are tubes or small lamellae connected by joints (branch points) and are numbered (six total) in the control crista for illustration. **I**: A partial remodeling of Bcl-xL/Lead mitochondria produced more thicker, tubular cristae (examples shown) than found in the control mitochondria.

**Figure 8:**
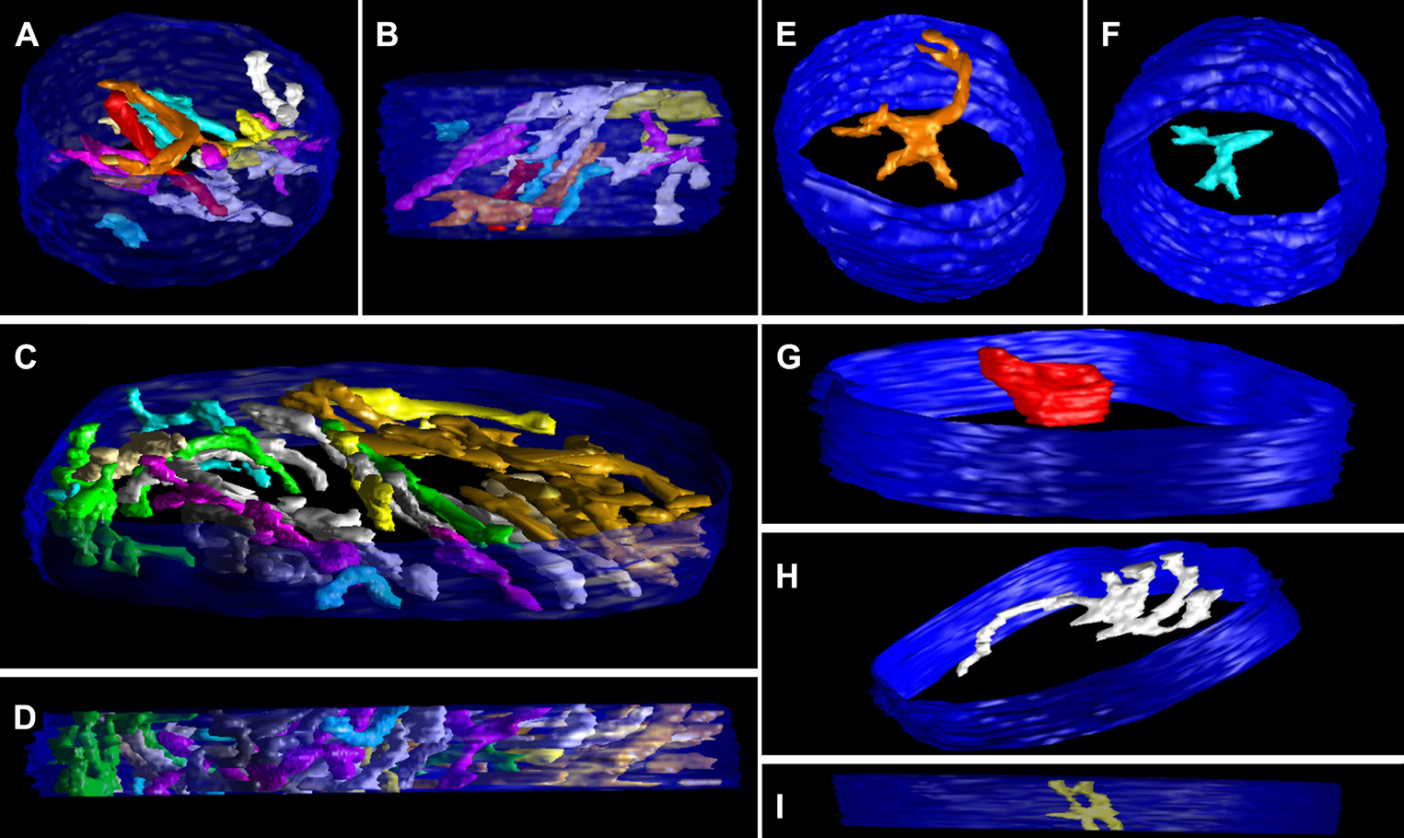
Lead-exposed pedicle mitochondria have more cristae with multiple segments than the controls or the lead-exposed spherule mitochondria. **A**: The oblique top and (**B**) side views show only those cristae with multiple segments (18 out of 177 cristae) in a control pedicle mitochondrion. The outer membrane is shown in blue. **C**: The oblique top and (**D**) side views show only cristae with multiple segments (33 out of 68) in a lead-exposed pedicle mitochondrion. Note the much greater volume of mitochondrion occupied by cristae with multiple segments in the lead example. Examples of typical cristae with multiple segments in the (**E**) control and (**F**) lead mitochondria. **G**: As is common with the lamellar cristae in the control mitochondria, they are small and do not extend far across the volume. Lamellar cristae are much rarer in lead-exposed mitochondria, whereas branched cristae with tubular segments are common. **H**: This is an example with a long tube. **I**: Branched cristae are equally common in Bcl-xL/Lead mitochondria.

One of the advantages of ET is the ability to examine the size, shape, constriction, connectivity, and branching of cristae as they extend through the mitochondrial volume. Figure 2, Figure 4, Figure 5, and Figure 6 show the full complement of cristae in each mitochondrion, which illustrates their packing density and arrangement. In Figure 7 (spherules) and Figure 8 (pedicles), a subset of cristae with multiple segments is presented to provide representative examples of lead-induced remodeling in connectivity, branching, and size among the crista segments. In the controls, about 20% of the spherule cristae (Figure 3C, right panel; Figure 7A,B) and the pedicle cristae (Figure 3F, right panel; Figure 8A,B) are composed of multiple cristae segments connected by branching points as illustrated in Figure 7G and Figure 8E. As noted, the lead-exposed spherule mitochondria have significantly more cristae with multiple segments (Figure 3C, right panel; Figure 7C,D,H; Table 2), and these cristae occupy a greater mitochondrial volume compared to that of the controls (Figure 7D versus 7B, respectively). The typical lamellar cristae in the control (Figure 7E) and lead (Figure 7F) spherule mitochondria are small, do not extend far across the volume, and are not structurally different. A side view in Figure 7I reveals a partial remodeling of the cristae in the Bcl-xL/lead spherule mitochondria. Although the fraction of the cristae with multiple segments in the Bcl-xL/lead spherules is not different from the controls (Figure 3C, right panel; Table 2), their cristae are thicker and have increased cristae surface relative to the lead mitochondria (Figure 3A, left panel; Figure 7D), albeit still not equivalent to that in the controls (Figure 3A, left panel; Figure 7B; Table 2). The cone pedicle mitochondria in lead-exposed mice also have significantly more cristae with multiple tubular segments (Figure 3F, right panel; Figure 8C,D,F; Table 2) and branched cristae (Figure 7H), and these occupy more of the mitochondrial volume compared to that of the controls (Figure 8D versus 8B, respectively). Lamellar cristae are much rarer in the lead-exposed pedicle mitochondria (Figure 7G) than in the controls. Side views of the Bcl-xL/lead pedicle revealed that the branched cristae are similar to those in the lead pedicle mitochondria (Figure 8I).

### Photoreceptor and synaptic terminal oxygen consumption

**Figure 9:**
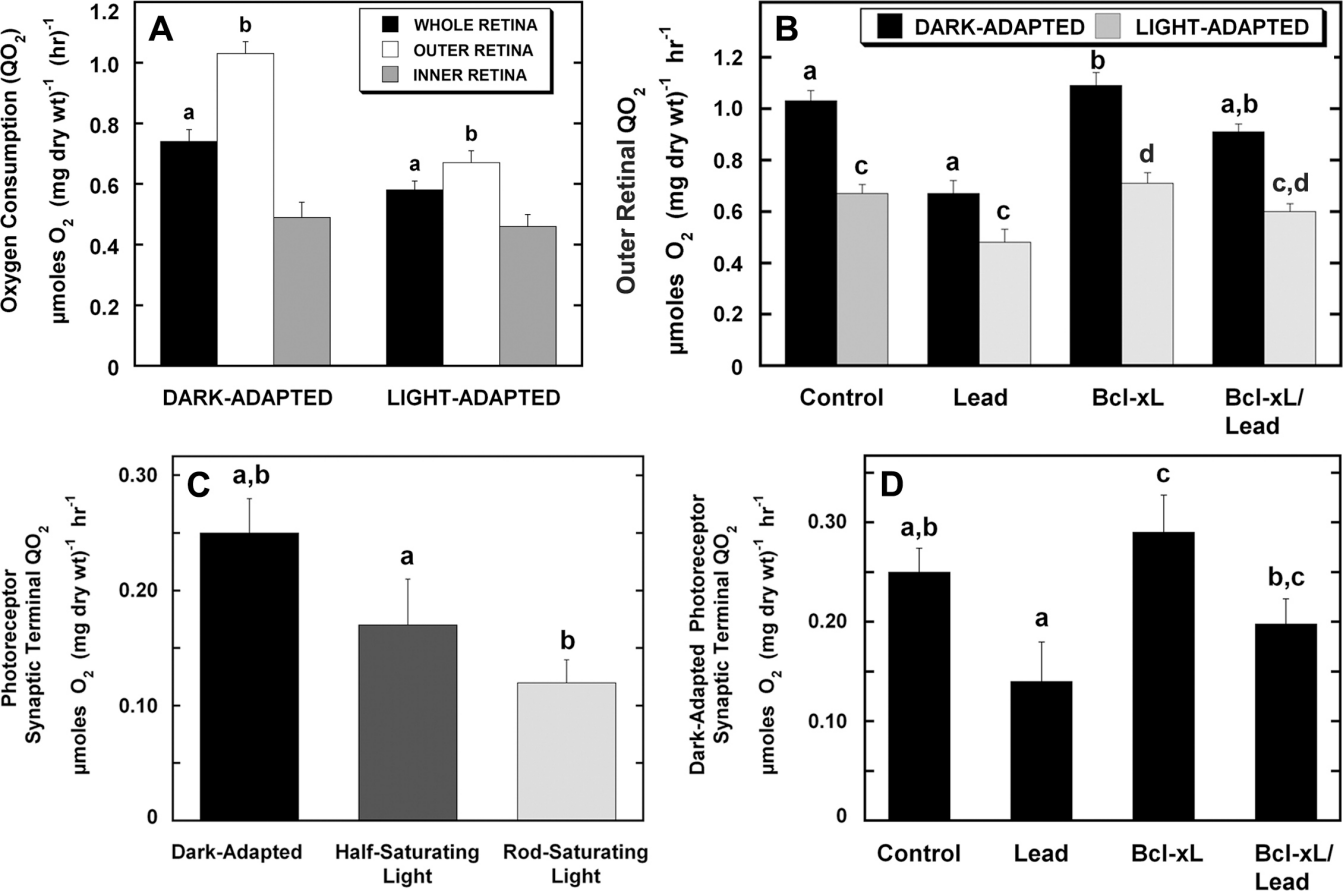
Lead decreases photoreceptor and synaptic terminal oxygen consumption (QO_2_) that is partially blocked by Bcl-xL overexpression. **A**: The QO_2_ in dark- and light-adapted whole retina, outer retina, and inner retina of adult control mice. Light decreased whole and outer retinal QO_2_. To determine QO_2_ in the outer (photoreceptors) and inner retina, the outer retina was pharmacologically isolated from the inner retina using 2-amino-4-phosphonobutyric acid (APB), 6-cyano-7-nitroqunioxaline-2,3-dione (CNQX) or 2,3-dihydroxy-6-nitro-7-sulfamoylbenzo-quinoxaline (DNQX), and D(-)-2-amino-5-phosphovalerate (APV) as described under Methods. **B**: Outer retinal QO_2_ in dark- and light-adapted control, Lead, Bcl-xL and Bcl-xL/Lead adult retinas. Lead decreased dark- and light-adapted outer retinal QO_2_, which were partially recovered in the Bcl-xL mice. **C**: Photoreceptor synaptic terminal QO_2_ in control dark-adapted retinas and retinas stimulated with half-saturating and rod-saturating light. Photoreceptor synaptic terminal QO_2_ was determined by adding CoCl_2_, nifedipine, or both to the outer retinal pharmacological buffer as described under Methods. **D**: Dark-adapted photoreceptor synaptic terminal QO_2_ in control, Lead, Bcl-xL, and Bcl-xL/Lead adult retinas. Lead decreased dark-adapted photoreceptor synaptic terminal QO_2_ that was partially prevented in the Bcl-xL mice. Values represent mean±SEM measurements from four to seven different mice from different litters per treatment. Values sharing the same superscript differed from each other at p<0.05.

The cristae surface/mitochondrial surface area ratio was significantly greater in the control spherule than pedicle mitochondria and was dynamically and differentially modified in the rod and cone synaptic mitochondria of the lead and Bcl-xL/lead adult mice (Figure 3A,D, left panels). To assess the long-term functional consequences of these prominent structural alterations related to ATP synthesizing capacity [70], four sets of new QO_2_ experiments were conducted in P60-P70 mice (Figure 9). First, the outer (photoreceptor) and inner retinal QO_2_ in the dark- and light-adapted isolated whole control retinas were examined using a more selective pharmacological isolation procedure than previously employed [29,60,61]. Second, the dark- and light-adapted outer retinal QO_2_ in all four groups was determined. Third, a procedure for measuring the photoreceptor synaptic terminal QO_2_ was developed and validated. Fourth, the dark-adapted photoreceptor synaptic terminal QO_2_ in all four groups was determined.

The dark-adapted control whole retinal QO_2_ was 0.74 µmole O_2_ mg dry wt^−1^ hr^−1^, and it decreased 22% during light adaptation (Figure 9A), consistent with our previous results [29,60,61]. The new pharmacological buffer significantly increased the sensitivity of the outer retinal QO_2_ measurement, such that the outer to inner retinal QO_2_ ratio was 2.1-fold higher with the new buffer compared to 1.8-fold with the older buffer [60]. In the controls, the dark-adapted outer retinal QO_2_ decreased 35% during light adaptation, whereas the inner retinal QO_2_ was unchanged, consistent with findings in isolated whole retina, isolated rod photoreceptors, and in situ QO_2_ measurements in mouse and rat retinas [29,59,60,76]. Figure 9B shows that lead significantly decreased the dark-adapted and light-adapted outer retinal QO_2_ by 35% and 28%, respectively, and that Bcl-xL/lead provided only partial protection, consistent with the ET substructural results for the spherule and pedicle mitochondrial cristae (Figure 3, Table 2). Since Bcl-xL overexpression blocked the rod-selective apoptosis initiated by RIS mitochondria [29], we reasoned that the persistent QO_2_ deficits in the Bcl-xL/lead retinas predominantly resulted from remodeled spherule mitochondria (Figure 3A, Figure 6A-D, and Figure 7I). To isolate and assess their function, a photoreceptor synaptic terminal QO_2_ method was developed by adding an L-type Ca^2+^ channel blocker, CoCl_2_, and/or nifedipine to the new buffer. The results in Figure 9C reveal that about 25% of the dark-adapted outer retinal QO_2_ originated from their synaptic terminals. Similar results were obtained with CoCl_2_ alone and with nifedipine in the absence or presence of CoCl_2_, so the data were combined, as presented. Half- and rod-saturating light decreased the dark-adapted QO_2_ by 32% and 52%, respectively, consistent with the decreased demand for ATP synthesis and increased oxygen tension in photoreceptor synapses during light adaptation [1,60,76,77]. Figure 9D shows that lead significantly decreased the dark-adapted photoreceptor synaptic terminal QO_2_ by 44%, which accounted for about 33% of the lead-induced decrease in photoreceptor QO_2_. Relative to controls, Bcl-xL increased this synaptic QO_2_ measure by about 15%, consistent with the increased number of rods in mice with photoreceptor Bcl-xL overexpression [29] and increased the metabolic efficiency of synapses with Bcl-xL overexpression [78]. In the Bcl-xL/lead retinas, the dark-adapted photoreceptor synaptic terminal QO_2_ remained significantly decreased relative to controls (−20%), although there was a 42% recovery relative to lead, findings consistent with the substructural results.

## Discussion

Three novel findings related to photoreceptor synaptic mitochondria structure, function, and remodeling were obtained in adult wild-type and Bcl-xL transgenic mice following the low-level postnatal lead exposure model. First, the spherule and pedicle mitochondrial ultrastructure and cristae substructure were dynamically altered following lead exposure. This was unexpected since only RIS, but not CIS, mitochondria were affected during the rod-selective apoptosis in similarly lead-exposed mice [29]. Second, outer retinal (photoreceptor) QO_2_ and photoreceptor synaptic terminal QO_2_ were decreased following lead exposure, indicating persistent dysfunction in mitochondrial oxidative phosphorylation. Third, Bcl-xL overexpression provided only partial protection against the lead-induced spherule and pedicle mitochondrial structural changes, and decreases in the outer retinal and photoreceptor synaptic terminal QO_2_. These findings were unexpected since Bcl-xL overexpression blocked the rod-selective transient opening of the permeability transition pore, cytochrome c release, and subsequent Bax-mediated apoptosis in similarly lead-exposed mice [29].

Retinas from lead-exposed mice displayed complex and distinguishing patterns of the spherule and pedicle mitochondrial cristae and matrix damage and remodeling. Mitochondria in both synaptic terminals exhibited matrix swelling consistent with classical permeability transition and exhibited vesicular cristae formation. In comparison, matrix swelling was not observed in RIS mitochondria during/following in vitro or postnatal lead exposure that exhibited permeability transition and rod-selective apoptosis [28,29]. The lead-exposed spherule mitochondria, compared to the controls, had decreased cristae abundance, an increased number of segments per crista, a larger fraction of cristae with multiple segments, and no difference in the number of cristae per volume. Thus, the lead-induced remodeling of the spherule mitochondria produced smaller cristae with more branching. In the Bcl-xL/lead spherules, cristae abundance was still decreased. In marked contrast, the pedicle mitochondria in the lead-exposed mice had an increase in the abundance of their cristae and the CJ diameter increased, an apparent enigma since matrix swelling and increased CJ diameter are almost always accompanied by a decreased number of cristae [45,51,79]. The pedicle mitochondria in the lead-exposed mice also had an increased number of segments per crista and a larger fraction of cristae with multiple segments. Thus, the lead-induced remodeling of the pedicle mitochondria produced larger cristae with more branching and increased CJ diameter. In the Bcl-xL/lead pedicles, cristae vesiculations and increased abundance were prevented. Four fundamental questions arise from these observations. First, why and how does lead exposure oppositely regulate the abundance of cristae and cristae volume in spherules and pedicles? Second, how does lead exposure increase cristae branching and CJ diameter? Third, how do the structural alterations contribute to the decreased outer retinal and photoreceptor synaptic terminal QO_2_? Fourth, how does Bcl-xL differentially remodel the cristae in spherules and pedicles?

We postulate that the answer to the first question is related to different inherent neuroprotective capabilities of the phylogenetically older cones [80,81] to altered cristae membranes and thus impaired oxidative phosphorylation and QO_2_. We previously speculated that the cones’ higher ATP production and lower sensitivity to Ca^2+^ overload resulted from their greater number of mitochondria and overall cristae abundance [1,29,31]. In lead-exposed mice, the decrease in the spherule mitochondrial cristae surface and volume likely results from a Bax-mediated transient opening of the OMM as occurs in RIS mitochondria [29] since no change in CJ diameter was seen. During classic apoptotic neurodegeneration, neuronal mitochondria in different brain regions, the optic nerve, and the peripheral nerve undergo matrix swelling followed by a Bax-mediated loss of cristae and cytochrome c [45,51,82]. In contrast, the relative increase in the pedicle mitochondrial cristae surface and volume might result from increased fission and decreased fusion, as these changes in mitochondrial dynamics accompanied by an increase in CJ size are associated with various neurodegenerative disorders [82,83]. These reasoned hypotheses suggest at least three potential targets of lead, the cytosolic dynamin-related protein Drp1 and its OMM receptor Fis1 that mediate OMM fission, the IMM Opa1 protein complex that regulates IMM fusion, and the large GTPases mitofusin 1 and 2 (Mfn1 and Mfn2) that reside on and mediate OMM fusion [51,82,83]. Further work is needed to determine whether lead binds to or activates any of these proteins and/or activates proteases that cleave Opa1.

Two possible mechanisms underlie the lead-induced increase in spherule and pedicle cristae branching as well as increased CJ diameter in pedicles. Both phenomena may result from increased expression of the formation of CJ protein 1 (Fcj1), as Fcj1 overexpression increased cristae branching, enlarged the CJ diameter, increased CJ formation, and reduced the levels of the ATP synthase supercomplexes [84]. Alternatively, the increased branching might result from increased Drp1-mediated fission, as we observed small fragments released from mitochondria, to compensate for the damaged cristae membranes and impaired oxidative phosphorylation (i.e., decreased QO_2_) since oxidative phosphorylation proteins primarily reside on cristae membranes [70,85,86]. Moreover, some studies indicate that Opa1 can remodel cristae and increase CJ diameter independently of fusion [53,54].

The answer to the third question links the lead-induced substructural changes in photoreceptor synaptic mitochondria with the decreased outer retinal and photoreceptor synaptic terminal QO_2_. Moreover, the answer raises an important question about long-term synaptic dysfunction following a developmental insult. The retina has one of the highest rates of QO_2_ of any tissue, and dark-adapted photoreceptors have a twofold higher QO_2_ than the inner retina [60,76,87]. Moreover, photoreceptor ribbon synapses support continuous vesicle release/recycling and reuptake of glutamate that is modulated by light and regulated by Ca^2+^-mediated processes and the α3-high ouabain affinity Na^+^/K^+^-ATPase that requires high rates of mitochondrial ATP synthesis [1,4,61]. Since the synaptic mitochondrial protein and RNA turned over at least twice since the end of lead exposure [57] and the blood and retinal [Pb] were not different from controls in adult mice [29, this paper], these findings indicate that early postnatal lead exposure produced persistent dysfunction in rod synaptic mitochondrial oxidative phosphorylation as manifested by decreased dark-adapted synaptic terminal QO_2_. We reasoned that the lead-induced decrease in the spherule cristae surface/mitochondrial surface area ratio would result in decreased photoreceptor and synaptic terminal QO_2_, as 95% of the photoreceptors are rods [88,89]. The swirls inside or budded from lead-exposed spherule mitochondria may be caused by destabilization of ATP synthase dimers [90] and/or deficient cytochrome oxidase activity [91] further supporting the lead-induced decreases in outer retinal and photoreceptor synaptic terminal QO_2_. Lead might have inhibited the synthesis of heme containing mitochondrial enzyme cytochrome oxidase and thus decreased outer retinal and photoreceptor synaptic terminal QO_2_. However, this appears unlikely since the lead-induced inhibition of mitochondrial coproporphyrinogen oxidase and/or ferrochelatase does not occur below 100 µM Pb^2+^ [92-94], and the maximum concentration of Pb^2+^ in the blood and retina of our lead-exposed mice at P21 was about 1 µM Pb^2+^. Moreover, a lead-induced decrease in the expression of the OMM voltage-dependent anion channel [35,36] would decrease cellular ATP and likely contribute to decreased photoreceptor and synaptic terminal QO_2_. The lead-induced alterations in the mouse rod and cone synaptic terminal mitochondria structure and function likely underlie the persistent scotopic and mesopic retinal deficits (i.e., decreased ERG amplitudes, dark adaptation and flicker fusion) observed in lead-exposed children, developing monkeys and rodents, adult rodents, and occupational workers [reviewed in 14,15]. Moreover, the alterations indicate that the permanent cognitive and neurologic impairments observed following low-level pediatric lead exposure [30,95] result from synaptic dysfunction [reviewed in 33,34].

Fourth, Bcl-xL overexpression partially remodeled the lead spherule and pedicle cristae and partially protected against the lead-induced decreases in outer retina and photoreceptor synaptic QO_2_ decreases. As noted [29], Bcl-xL has pleiotropic antiapoptotic effects, and it protects cells from apoptosis triggered by Bax and Bak, Ca^2+^ overload, and reactive oxygen. In addition, recombinant Bcl-xL blocks tBid-induced apoptosis mediated by cytochrome c release and the disassembly/loss of Opa1 complexes [47]. Recently, novel functional roles of Bcl-2 and Bcl-xL were found. Bcl-2 and Bcl-xL overexpression enhanced the efficiency of neuronal energy metabolism via a direct interaction with mitochondrial F1F0 ATP synthase and increased mitochondrial oxygen consumption by regulating the cellular redox status [78,96]. It is not known which of these effects contributed to the differential remodeling in the lead-exposed spherules and pedicles. However, the decreases in the rod photoreceptor and synaptic QO_2_ in Bcl-xL/lead mice likely resulted from the decreased cristae abundance (ATP synthesizing activity [70]) as this was the only persistent structural change (see Table 2).

Synaptic mitochondria play a fundamental role in health, neurodegeneration, retinal degeneration, neurologic disease, and aging [1,97-102]. Inhibition of apoptosis by Bax deletion or overexpression of Bcl-2 or Bcl-xL does not prevent long-term synaptic neuropathology [98,103,104]. In this study, we observed that rod and cone synaptic mitochondria from Bcl-xL/lead mice with normal blood [Pb] and likely significant presynaptic mitochondrial turnover still exhibited structural and functional deficits. Although the mechanisms are unknown, the results are consistent with other studies showing that synaptic mitochondria are more sensitive to Ca^2+^ overload, oxidative stress, and ATP loss than non-synaptic mitochondria [97,98,102,105,106]. Our findings that Bcl-xL blocked the lead-induced rod-selective apoptosis, but did not protect against Ca^2+^ overload in rods [29], indicates that a similar mechanism likely occurs in rod spherule and cone pedicle mitochondria. If so, this suggests that differential handling of Ca^2+^ overload in photoreceptor synaptic mitochondria, relative to RIS and CIS mitochondria, increased their vulnerability to permeability transition, consistent with the above studies [105,106]. In summary, our findings combined with those of others indicate the clinical and scientific importance and relevance of examining for long-term synaptic dysfunction following injury or disease during development, and for developing effective therapeutic strategies and treatments that prevent synaptic degeneration in retinal and neurodegenerative disorders even if apoptosis is blocked.
